# Computational elements of natural vision

**DOI:** 10.1167/jov.25.12.4

**Published:** 2025-10-03

**Authors:** Constantin A. Rothkopf, Mary M. Hayhoe

**Affiliations:** 1 Centre for Cognitive Science & Institute of Psychology, Technical University of Darmstadt, Darmstadt, Germany; 2 Centre for Perceptual Systems, University of Texas at Austin, Austin, TX, USA

**Keywords:** natural tasks, sensorimotor behavior, computational model, perception and action, partially observable Markov decision process

## Abstract

Ultimately, human behavior needs to be understood in the context of natural everyday tasks. Over the last two decades, a number of observations of natural visually guided behavior have accumulated. These observations help define the functional demands placed on the visual system in a variety of tasks, but progress has been limited by the diversity of natural behavior and by the lack of unified theoretical structures to guide understanding of the underlying processes. In this article, we summarize some recent attempts that might provide a template for a more formal approach. This is possible because it has become clear that natural behavior has many regularities reflecting the underlying sensorimotor decisions. We first summarize these regularities and then show how simple visually guided behaviors can be well described by partially observable Markov decision processes. We give examples of how laboratory experiments can be designed to elicit the common elements of natural behavior and how such experiments afford control of the statistical structure of tasks, thereby allowing formal modeling. Finally, we suggest that a new exciting avenue using recently introduced inverse models may lead the way forward, as it recovers the intrinsic properties of human perception, cognition, and action, which are intertwined in natural behavior.

## Understanding the structure of natural sensorimotor behavior

In order to survive, sensory systems must provide organisms with information about the world that allows them to make good action choices. Historically, studying this problem has been addressed by breaking the problem down into simpler components and devising experimental paradigms that control or eliminate factors other than the one under consideration. Thus, traditionally human behavior has been compartmentalized into different cognitive faculties, such as perception, attention, memory, decision-making, and motor control, that are typically investigated separately. However, it is becoming increasingly clear that it is necessary to examine behavior in more natural contexts in order to better understand how even the most simple sensory decisions and action choices are made (Cisek & Pastor-Bernier, 2014; Krakauer, Ghazanfar, Gomez-Marin, MacIver, & Poeppel, 2017; Hunt et al., 2021; Yoo, Hayden, & Pearson, 2021; Miller et al., 2022; Fooken et al., 2023; Maselli et al., 2023; Cisek & Green, 2024). Under such circumstances, perception, attention, memory, decision-making, motor control, and learning interact in concert to achieve the behavioral goals of the organism. Therefore, the question arises about how to best study natural behavior: If perception, cognition, and action are intertwined under such circumstances, how can experiments be devised that investigate this interplay while maintaining some form of control?

Over the last two decades or so, particularly following the seminal work of Michael Land (Land & Lee, 1994; Land & Furneaux, 1997; Land, Mennie, & Rusted, 1999; Land & McLeod, 2000) and others (Ballard, Hayhoe, & Pelz, 1995; Epelboim et al., 1995; Land & Hayhoe, 2001; Hayhoe, Shrivastava, Mruczek, & Pelz, 2003), there have been a number of investigations of natural visually guided behavior. Observing natural behavior allows us to better define the problems that the visual system has to solve, as well as providing more precise descriptions of the visual stimuli incident on the retinas during active behaviors. Such observations have led to a number of insights (see Hayhoe, 2017, for a review). However, the diversity of natural behavior is huge and the domain unstructured. To gain insights into the underlying decision processes and ultimately the associated neural underpinnings, we need to develop a principled approach to the analysis of natural behavior. In this article, we review some recent investigations that provide a template for a more formal approach that allows generalization beyond a particular example behavior. First, based on the example behavior of navigating rugged terrain, we attempt to characterize a set of properties of natural visually guided actions that appear to be common to many different behaviors and contexts. We then proceed by describing how these properties can be understood within the framework of sequential decision-making under uncertainty. We follow this with an examination of some recent experiments that are designed to preserve the essential properties of natural behavior while maintaining experimental control of a particular variable. By fitting sequential decision models to the behavior, we can make stronger inferences about the underlying mechanisms. Finally, we provide an outlook to the potential of recent inverse modeling for the understanding of natural visual behavior.

### Observing and measuring natural behavior

An essential aspect in understanding natural behavior, say in unconstrained navigation of a natural, rugged environment, is the need for complete data sets of the behavior. Eye movements constantly change the retinal images, as do body movements, and in turn, the retinal images provide the information required for actions. Consequently, an understanding of even simple actions in the natural world requires that we monitor both the actions and the visual input. The technology for monitoring gaze and body during natural behavior is improved and easier to use, and computer vision techniques now allow reconstruction of three-dimensional (3D) representations of the visual scene. Together with gaze and body data, this allows specification of the visual information that is used in action decisions. In recent work, Muller et al. (2024) integrated gaze and body tracking with a numerical representation of the visual environment, obtained using photogrammetry algorithms that reconstruct the 3D terrain structure, together with RGB values. This allowed description of the way the image changes with body movements and analysis of the visual information used for foothold choices. We will use the observations made in this study to provide intuitions and give examples of common elements of natural behavior, demonstrating the complexity of the processes involved in a very basic visually guided behavior.

What are aspects of behavior in a natural task such as unconstrained navigation of a rugged terrain? First, gaze is frequently directed toward distant locations in flat terrain. We can think of this as reflecting the use of a memory-based *prior* that is sufficient to guide locomotion with infrequent visual updates for reducing uncertainty about the walking surface close to the observer. As the path becomes more rugged, gaze is increasingly directed at the ground close to the walker, reflecting increased need for information about the variable ground surface (Matthis, Yates, & Hayhoe, 2018; Muller et al., 2023). This change in gaze behavior is quite regular and sensitive to the visual properties of the surface. Walkers slow down and take shorter steps, reflecting a need for more time to *reduce visual uncertainty* for choosing footholds, together with an increase in the cost of *motor errors* that now needs to be traded off against *energetic costs* (Matthis, Yates, & Hayhoe, 2018; Bonnen et al., 2021). These adjustments approximate a strategy of looking a constant time ahead, averaging about 1.5 seconds, suggesting that visual working memory decay might determine how far ahead walkers can look. Not only does gaze location indicate the need for integration of visual information over time, but chosen paths reflect motor plans that extend at least five steps ahead (Muller et al., 2024). Subjects choose paths where the average height change in a segment of approximately five steps is less than neighboring possible paths, indicating *planning of step sequences*. The preference for flatter paths is presumably driven by both the energetic cost of height irregularities and the increased stability associated with smaller height changes. Circuitous paths also incur energetic cost, and we found that walkers chose longer paths when the direct paths involved greater height changes. This *trade-off* depended on leg length, indicating that walkers have remarkably good estimates of the costs that are involved in the trade-off, which are specific to their own bodies and their individual motor variability. This suggests that walkers use well-defined internal models for state estimation and control during walking. Thus, locomotion likely reflects a tight *interplay between perception and action*, where the acquisition of task-relevant information is modulated by the availability of prior representation in a manner determined by sensory and motor uncertainty and varying costs. Both gaze and walking behavior reveal the need to hold visual information in working memory and to plan action sequences. Thus, locomotion is an elegantly orchestrated decision process that unfolds in time and space.

### Common elements of natural visually guided behavior

Considering the above example of navigating rugged terrain, we proceed by first identifying common elements of natural visually guided behavior. The goal is to find common motifs of behavior and to show in subsequent sections of this article how these elements of natural behavior can be understood and modeled in a formal setting.

#### Forming priors and beliefs about world state

Vision is used to choose good actions. How does this happen? It begins with an evaluation of the state of the world relevant for ongoing behavior. However, the true state of the world is always unknown, such as one’s own exact walking speed, the distance and time to reach a foothold in rugged terrain, the exact moment when a traffic light will switch, or the path of another pedestrian. Consequently, we must form beliefs on the basis of incomplete, uncertain, ambiguous, and noisy sensory measurements. It is commonly accepted that such an evaluation is computationally well described by Bayesian inference (Knill & Richards, 1996; Kersten, Mamassian, & Yuille, 2004). Sensory data are combined with information stored in memory, formalized as a prior belief, to compute an estimate of the world state, formalized as the posterior belief. This posterior probability estimate of the world state, given the data and the prior, can be more or less noisy depending on the precision of the memory and of the sensory data. Laboratory experiments have shown that participants can *learn priors for guiding actions*. For example, the light from the above prior can be altered through repeated exposure to visuo-haptic scenes (Adams, Graf, & Ernst, 2004), and participants can learn priors over distances to targets in path integration experiments, which they subsequently use in their navigation behavior (Petzschner & Glasauer, 2011). Early studies by He and Kowler (1989) showed that short latency saccades to targets are not simply reflexive but take prior knowledge into account. Importantly, it appears that the uncertainty associated with the posterior is preserved in the neural computation (Walker, Cotton, Ma, & Tolias, 2020) and ultimately reflected in actions (Ma & Jazayeri, 2014). In a simple example, reaction times reflect the uncertainty involved in a perceptual decision and indeed can be used as a measure of detectability (Lappin & Disch, 1972).

#### Task goals, costs and rewards, and motor noise

Given a belief about world state and its associated uncertainty, a number of considerations involved in the choice of the action can be described in the context of Bayesian decision theory (Trommershäuser, Maloney, & Landy, 2003; Trommershäuser, Maloney, & Landy, 2008). Most critically, action decisions are made to serve behavioral goals. Looking at targets may involve different costs depending on whether the behavioral goal is to inspect locations visually or to tap them (Epelboim et al., 1995). This is a pervasive constraint in understanding natural behavior. One can think of all visual computations as satisfying some particular goal for the organism or as being a component that allows satisfaction of some larger goal. Task goals define the sensory data required for the posterior. In common laboratory experiments, subjects are instructed to carry out a particular task or are incentivized to pursue a goal (e.g., with monetary rewards). This is different from natural behavior. For example, walking in rugged terrain requires locating the position and height of a foothold, which might be determined by visual search or from spatial memory if the environment is familiar. The goal also identifies the cost and benefits associated with a particular action choice. Foot placement needs to take into account the cost of tripping, for example. Humans also minimize energetic cost by adopting a preferred gait determined by their own biomechanics (Kuo, Donelan, & Ruina, 2005; Selinger, O’Connor, Wong, & Donelan, 2015; Finley, Bastian, & Gottschall, 2013; Lee & Harris, 2018). Gait parameters might be modified, however, in the context of another constraint such as needing to get to class on time. These behavioral adjustments to the time-varying costs and benefits are driven by the pervasive operation of the neural reward machinery (Schultz, 2015). As with the evaluation of environmental state, action decisions must also take into account noise in the motor system, and this, too, is an integral part of the computation that determines the action choices. Human subjects can learn to adjust their actions, such as fast pointing movements when the variability of action outcomes and the associated rewards are manipulated experimentally (Seydell, McCann, Trommershäuser, & Knill, 2008). Thus, walking speed will take into account both the ruggedness of terrain and the probability of tripping when walking quickly.

#### Coordinating perception and action over time

The final pervasive element in natural behavior, unlike many laboratory paradigms, is that it is a process that plays out at least over time scales of several seconds or minutes. Therefore, a change in gaze or body movement changes the sensory input and evaluation of the state of the world, which in turn determines the next action decision. Thus, the current posterior, together with knowledge of the behavioral context, repeatedly becomes the new prior for the next action decision (Lee, Ortega, & Stocker, 2014). For example, in visual search, it may be better to look in between two potential target locations instead of at the currently most promising target (Najemnik & Geisler, 2005), because this best allows disambiguating potential targets and selecting the next eye movement. If a good internal model or a stable memory is available, *multistep planning* of gaze movements becomes particularly advantageous. Early studies by Zingale and Kowler (1987) found behavioral evidence for the planning of gaze sequences and allowed characterizing the associated behavioral trade-offs (Araujo, Kowler, & Pavel, 2001). Accordingly, adjusting sequences of gaze movements when walking in rugged terrain to looking ahead at a constant time is a clear indication of planning. As we will address below, action plans therefore require internal perceptual models that allow prediction over times scales of seconds. Visual working memory is an essential component of this process in order to maintain such information.

A further consequence of the extended time horizon in natural behavior is that a task goal is often reached at the end of a sequence of actions, so that such tasks fundamentally involve both prediction of a future state and planning sequences of actions. The reason is that carrying out just the next best action (i.e., acting greedily) may not help in achieving the long-term goal. Thus, natural behavior involves sequences of perception, cognition, and action, which all influence each other reciprocally. In this scenario, sequentially estimating the world state interacts with sequentially carrying out actions to achieve the task goal and, therefore, the associated costs and benefits. Because of these behavioral costs, just making an eye movement to update one’s belief and reduce uncertainty about the world state trades off with the costs or rewards associated with the task goal. Thus, natural tasks require *multistep planning of both perception and action*. Because the true world state is ambiguous, uncertain, and noisy, planning gaze and body movements takes place relative to the current internal, uncertain belief. Navigating rugged terrain therefore can be understood as *path-planning under uncertainty* relative to the current belief (i.e., the mental map of the environment). A further complication that must be taken into account here is that vision, eye movements, and body movements all function at different time scales, with their associated delays and different uncertainties, so prediction of the world state requires internal models of the body that take these time constants into account. This may involve a trade-off between the time for accumulating visual evidence and the duration of a movement (Battaglia & Schrater, 2007) but can also adjust the timing between gaze shifts and hand movements, depending on the difficulty of visual tasks (Sims, Jacobs, & Knill, 2011). In natural behavior, for example, it is most energetically efficient, when approaching an obstacle in the path, to increase walking speed prior to stepping over the obstacle and then to slow down subsequently (Darici & Kuo, 2023). Such adaptations of *flexible strategies* are ubiquitous in natural behavior, as is the coordinated adjustment of step size and distance of looking ahead when navigating rugged terrains.

Taken together, even the simplest actions involve both long-term and short-term memory, an evaluation of sensory and motor uncertainties and costs, and planning that takes place over time scales of seconds, in the context of action sequences. Conceptualizing perception and action in trial-based experiments may permit the assumption that a single stimulus leads to a percept followed by a decision-making process and finally a motor control process, in an essentially feed-forward manner. However, this is not the case in sequential behavior, because an action in a sequence changes the current belief about the state of the world by reducing perceptual or motor uncertainty, and it also brings the subject closer to achieving the goal (Yang, Wolpert, & Lengyel, 2016). Thus, perception and action become intertwined because they both affect the current belief of an organism about the expected goal achievement in the long run. Therefore, it is this statistical structure that fundamentally determines the “naturalness” of a task, we argue here, as this is what gives rise to the elements of natural behavior.

These common elements of natural sequential behavior have fundamental consequences for developing a more formal approach. Bayesian decision theory captures only a single action decision. Simply picking the action that looks best at each moment, a policy called “myopic” or “greedy,” is in general highly suboptimal in extended tasks involving multiple actions (Russell & Norvig, 2010; Mattar & Lengyel, 2022). Instead, as we will discuss, sequential perception and action under perceptual uncertainty, behavioral variability, and internal model uncertainty constitute a fundamentally different computational problem, which is better described as a partially observable Markov decision processes, or POMDP (Astrom, 1965; Kaelbling, Littman, & Cassandra, 1998), and the evolution of decisions across time can only be addressed through planning (Kaelbling, Littman, & Cassandra, 1998; Kochenderfer, 2015). Computationally, planning is necessary in order to achieve a behavioral goal if this involves not only a single action but also a sequence of actions. Behavioral sequences are planned when “deciding on a course of action by considering possible future situations before they are actually experienced” (Sutton & Barto, 2018). Hence, planning is defined as taking future situations, observations, or rewards into consideration during current action selection. Because of this fundamental difference between natural tasks and tasks involving single short-duration trials with single decisions, it is important to study how perception, cognition, and action are orchestrated in naturalistic extended behavior.

## Theory-driven approaches to understanding natural behavior

The regularities in natural behavior described above and the possibility of more complete data sets nourish the hope that more formal computational approaches to understanding extended, sequential, naturalistic visually guided behavior may become feasible. And indeed, large behavioral data sets in combination with data-driven computational methods have started to yield new insights into human decision-making and navigation abilities (Wu, Schulz, Speekenbrink, Nelson, & Meder, 2018; Peterson, Bourgin, Agrawal, Reichman, & Griffiths, 2021; Coutrot et al., 2022). However, understanding human sensorimotor behavior in natural sequential tasks through theory-driven computational models is a formidable task. Such a formal treatment requires not only measuring the subject’s behavior but also specifying the properties of the task itself. This is a fundamental difference from many data-driven computational approaches. Using the example of the previous section, a formal model of walkers’ gaze selection requires knowing participants’ subjective uncertainty about the terrain, the uncertainty reduction resulting from their eye movements, their internal model’s uncertainty about the foot placement, and their subjective utilities and internal costs in the task. But how can these quantities be recovered? What we want to argue for here is that a first approach is to devise laboratory experiments that are characterized by the common elements of natural tasks but also allow for tight control of the above quantities. This requires computational models that incorporate a perceptual, internal model and action uncertainties sequentially. Finally, we will argue for the use of inverse models, which help estimate the unknown quantities such as the internal costs, action variability, or perceptual priors.

**Figure 1.**
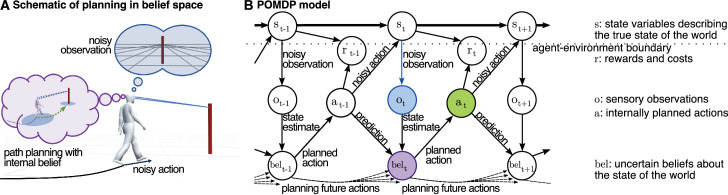
Illustration of a POMDP. (A) In a navigation task, the state variables fully describe the momentary physical configuration, including the spatial positions and velocities of objects, including the subject as well as the subject’s true looking direction. However, the subject only has access to ambiguous, noisy, and uncertain sensory observations. The sequence of sensory observations, together with internal model priors, gives rise to the internal, subjective belief (i.e., the internal sense of space). Planning of an action is relative to this internal belief, and motor variability gives rise to the actual noisy action in the world. (B) The corresponding formalization of a POMDP as a Bayesian network. Circles correspond to random variables, and arrows correspond to statistical dependencies.

In what follows, we attempt to describe this approach in more detail. We describe several recent studies in which paradigms were specifically designed such that their task structure elicits the common elements of natural visually guided behavior and at the same time allows for computational analyses of the behavior. We show how laboratory experiments can have a task structure that leads to naturalistic behavior while allowing for the tight control of the task-relevant probabilistic quantities and how some of the subject’s internal parameters can be estimated as a result. We will sketch multiple ways of how to capitalize on these approaches and what future research questions may be addressed with this approach. A central role in all these studies will be filled by POMDPs (Astrom, 1965; Kaelbling, Littman, & Cassandra, 1998) that formalize the different aspects of sequential decisions under uncertainty. This class of models can be seen as an extension of Bayesian decision theory from single decisions to sequential decisions. In Bayesian decision theory, we are uncertain about the outcome of a single decision. One way of formalizing this is to assume that the decision-maker has an internal belief, the so-called belief state, which characterizes their belief about the outcome of a decision. Alternatively, POMDPs can also be understood as extensions of Markov decision processes (MDPs; Dayan & Daw, 2008; Sutton & Barto, 2018), which describe the common situation in a reinforcement learning task. However, the reinforcement learning setting commonly assumes that the decision-maker has no perceptual uncertainty about the state (e.g., as in a game of chess or Go, where the state of the game is fully described by the position of the game pieces on the board). This is fundamentally different from the situation encountered by organisms, including humans, as sensory organs provide uncertain, ambiguous, and noisy measurements about the state of the world so that only a belief over the state of the world is accessible. Indeed, POMDPs (see Figure 1) can accommodate perceptual uncertainty by connecting the state of the world *s* and perceptual observations *o*, which form the belief *bel*, actions *a*, costs and rewards *r*, internal model uncertainty, action outcome variability, and both external rewards as well as internal costs over time, thereby covering the common elements of natural visually guided behavior.

Note that in this article, we do not attempt to review and discuss other approaches to predicting sequential behavior, for example, approaches (Chen, Jiang, & Zhao, 2021; Mondal et al., 2023; Yang et al., 2024) that use supervised learning of gaze sequences on large data sets and were able to successfully predict scan paths in several different behavioral contexts, or work that uses inverse reinforcement learning for visual search in pixel space (Mathe & Sminchisescu, 2013; Jiang et al., 2016; Yang et al., 2020). This work deserves a more careful discussion than we can include in the current context. We have focused on POMDPs as normative models in the current article as these models can explicitly represent the computational-level characteristics of natural tasks that we describe above, such as noisy observations, internal model uncertainty, cost and benefits, action variability, planning, and prediction, that relate more easily to behavior and to the way we conceptualize experiments. By devising experiments that recognize the potential role of these factors, we hope that our results will generalize more readily, meaning that parameters such as those describing sensory noise can directly be compared across participants, conditions, and tasks. The models then allow representation of these particular decision factors in an interpretable way. Our goal was simply to make more explicit the way that POMDP models could be used to devise paradigms that might allow more controlled investigation of natural behavior and to provide some examples of how this might be achieved in the future. There are a variety of other noncomputational approaches to developing a better understanding of natural behavior that we do not cover in this article. A number of these approaches have been described in a companion article by Goettker, Powell, and Hayhoe (2025). In the present article, our goal was to explain in more depth how the experiments and associated models that we review here might be useful. We expand on how the paradigms and models can be used at the end of each experiment in the sections labeled “The value of the computational model.” In those sections, we discuss how modifications of the paradigms can be used to estimate internal variables and predict behavior. This then leads into the inverse modeling described in the last section.

### Learning priors for guiding actions

First we address the issue of priors. In many situations, priors reflect the history of experience over developmental and perhaps evolutionary time scales. However, it has become increasingly clear that human perceptual systems are sensitive to changes in environmental statistics that occur on much shorter time scales (Jovancevic-Misic & Hayhoe, 2009). This allows us to observe situations where the priors are learned. We have also mentioned the central importance of prediction in evaluating an environmental state, and the deployment of gaze in time is a useful way to reveal these predictions. A number of studies suggest sophisticated temporal control of eye movements coordinated with task-relevant events in natural behavior (Johansson, Westling, Bäckström, & Flanagan, 2001; Hayhoe et al., 2003; Hayhoe & Ballard, 2005; Jovancevic-Misic & Hayhoe, 2009; Land & Tatler, 2009). A common observation is that gaze is often predictive of both physical events (Diaz, Cooper, Rothkopf, & Hayhoe, 2013) and events caused by other people (Flanagan & Johansson, 2003), precisely timed relative to task events, and this temporal gaze coordination must be learned. However, a precise quantitative characterization of the temporal structure and its associated uncertainty of events unfolding in natural behavior has been difficult to come by, because it is difficult to measure quantitatively (e.g., the uncertainty of a cricket player about the position, speed and bounce point of an approaching ball; Land & McLeod, 2000). An alternative route is to instead control the temporal statistical structure in a laboratory experiment by generating events from a statistical generative model and measure how progress in learning changes behavior. This allows for the computational analysis of gaze behavior and participants’ learning progress.

**Figure 2.**
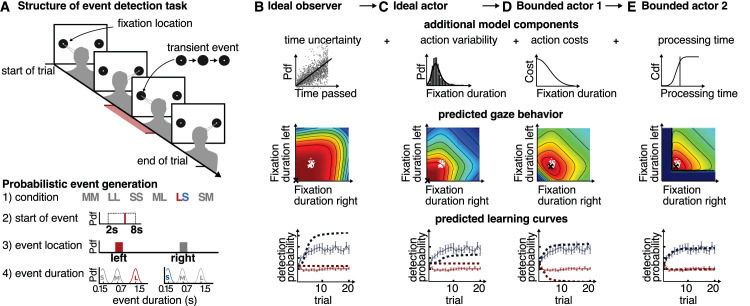
Models of temporal gaze selection. (A) Structure of the event detection task with the probabilistic event generation. In the temporal event detection task subjects are instructed to detect a single transient event per trial, which can happen at one of two fixed spatial locations. Over trials, subjects could learn the probabilistically generated event durations and gradually shifted their observation durations to better detect the events. (B) The ideal observer is based on the perceptual uncertainty about the amount of time that has passed, a Weber law. Note that an action such as a gaze shift is not part of the ideal observer, which only performs inference. (C) The ideal actor has the goal of increasing the probability of detecting the event under perceptual uncertainty but additionally takes its motor variability (i.e., the variability in fixation durations) into account. (D) The bounded actor takes into account the cost of a gaze shift (i.e., it internally trades off the reward of detecting an event in the experiment with the cost of making one additional eye movement). (E) The final model is additionally bounded by the biological constraint of the minimal duration of a fixation. Each row shows the additional model component and how it affects the predicted fixation durations at the two event locations and the predicted learning curves over trials.

To investigate how people can acquire knowledge about environmental event statistics and use them as priors to guide visual behavior, Hoppe and Rothkopf (2016) devised a temporal event detection task (Figure 2A). Subjects were instructed to detect probabilistically occurring events at one of two spatial locations. Within blocks of 20 trials each, the event durations at the two locations were each held constant. At the beginning of a block, participants spent equal observation times monitoring the two targets for possible occurrences of an event. Over trials, subjects learned about the two event durations and progressively looked longer at the location with the shorter event duration. This implies that internal beliefs about the event durations were learned and subsequently used to adjust the perceptual strategy of looking for future events. An important feature of the task is that the informativeness of spatial locations changes moment by moment, which corresponds more to the situation in natural, dynamic environments and not fixation by fixation. Under such circumstances, optimality of behavior is not exclusively evaluated with respect to a single, next gaze shift, but instead is driven by a complex sequence of behavioral decisions based on uncertain and noisy sensory measurements and the passage of time. In this respect, the temporal event detection task (Hoppe & Rothkopf, 2016) is much closer to natural vision than experiments with static displays. Thus, taken together, the behavioral data in this experiment clearly demonstrated that participants first attended to the two locations to learn about the event statistics and then gradually shifted to using the learned event statistics as priors to guide when to attend to the two locations. However, one peculiarity in the behavioral data was that while performance of participants improved across trials in the difficult conditions (i.e., with shorter event durations), participants’ performance over trials slightly worsened in the easier conditions (i.e., with longer event durations).

To quantitatively describe and explain participants’ gaze-switching behavior in response to the event statistics, Hoppe and Rothkopf (2016) asked which behavioral components needed to be included in a computational model to capture the empirical data well enough (see Figure 2B–E). Because the event durations in this experiment were generated according to a probabilistic model and under full control by the experimental design, it was possible to develop a probabilistic model of participants’ behavior. Overall, the behavioral data were best explained by a computational model that includes perceptual uncertainty, including the well-known Weber law for time estimation (Gibbon, 1977), the known heavy tail distribution of fixation duration variability (Ratcliff, 1979), a known minimal perceptual processing time (Remington, Wu, & Pashler, 2011), and, crucially, an intrinsic cognitive cost for each additional gaze switch (see Figure 2E). This intrinsic cost could succinctly quantify and explain the peculiar performance behavior of participants across different conditions: On average, subjects traded off the event detection rate with the behavioral costs of carrying out eye movements for acquiring additional information. This cost was stable at about a 1.55% detection probability for doing one less gaze shift across conditions and participants. Thus, the computational model was necessary to reveal the intricate trade-off between goal achievement (i.e., detecting events) and information acquisition (i.e., switching gaze one additional time). This trade-off between task achievement and internal costs for cognition and behavior can be regarded as a form of resource-rational (Simon, 1955; Anderson, 1991; Gershman, Horvitz, & Tenenbaum, 2015) sensorimotor behavior. Remarkably, based on this rational bounded actor model, the time course of learning the gaze strategies across trials is fully explained without further assumptions or free parameters by an optimal Bayesian learner, where the uncertainty in participants’ beliefs arises from humans’ characteristic uncertainty about the time passed (i.e., the Weber law for time estimation). Taken together, these findings established computationally that the human visual system is highly efficient in learning priors for temporal regularities in the environment and that it can use these regularities to control the timing of eye movements to detect behaviorally relevant events. This particular analysis was made possible by fixing the spatial locations and using simple geometric shapes, but otherwise allowing subjects to simply perform that task in an unrestricted manner.

#### The value of the computational model

In this study, a controlled temporal event detection task was devised, starting from a computational model involving common elements of natural visually guided behavior. The value of the model lies in the fact that participants’ gaze-switching behavior is quantitatively described and explained in terms of interpretable quantities. Thus, while observation of behavior in natural tasks suggested that people can learn priors to adjust the timing of gaze, here the timing could be computed from experimental variables. By systematically adding different model components, it was possible to successively augment the model to find which components were necessary and sufficient to capture observer behavior. This included subjective, internal cognitive costs for obtaining new information with an additional gaze switch. While in this experiment, the cost of vision being suppressed during a saccade is absorbed in the probability of missing an event, the very real cost of suppressing vision during a blink has been also captured in a separate study (Hoppe, Helfmann, & Rothkopf, 2018). Note that these are fundamental departures from ideal observer analysis, as participants’ behavior is not evaluated relative to the researcher’s conceptualization of what participants ought to do in the experiment. Instead of concluding that participants were “suboptimal” as performance decreased over trials in the easy conditions or reporting an “efficiency” value, the computational model allowed for an intrinsic cost of acquiring more information with a gaze switch. Accordingly, this establishes a resource-rational explanation of participants’ sensorimotor behavior (Simon, 1955; Anderson, 1991; Gershman, Horvitz, & Tenenbaum, 2015). The computational model allowed estimation of this quantity from participants’ behavior, and in this case, the cost for a single gaze switch was estimated to be 1.55% detection probability across conditions. Thus, the computational model was necessary to find this internal behavioral trade-off, and given this trade-off, participants’ learning curves were predicted without any further assumptions, thereby establishing people’s near-optimal learning of priors for guiding actions. This constitutes further support for the validity of the computational model beyond statistical model comparison. Based on this model, participants’ learning rates were predicted without introducing any additional parameters.

Importantly, this model now makes it possible to devise further experiments that can systematically quantify the unobserved quantities describing behavior, particularly because all model components are readily interpretable. As an example, the cost for a gaze switch could be characterized systematically and quantitatively as a function of the spatial separation or the visibility of the two event locations. Similarly, the cognitive cost of a gaze switch could be characterized by giving subjects a concurrent cognitive load. Thus, by using a controlled paradigm, which nonetheless includes multiple aspects of natural sequential decision-making, it becomes possible to estimate internal quantities that may remain stable across a variety of contexts.

### Multistep planning of perception and action

As we have described, natural sensorimotor behavior unfolds over time scales of several seconds or minutes. Thus, natural behavior inherently involves the coordination of perception-action sequences, which is computationally equivalent to planning. Experimentally, several studies had found evidence for planning of gaze sequence selection (Zingale & Kowler, 1987; Araujo et al., 2001). Perhaps the most elementary task to test whether natural sequential decision-making under uncertainty can be quantitatively predicted by a computational model as described above is the situation in which subjects are able to plan a sequence of just two eye movements. To computationally investigate planning of gaze sequences, Hoppe and Rothkopf (2019) developed a laboratory task whose statistical structure allowed computing different planning strategies within the POMDP framework.

**Figure 3.**
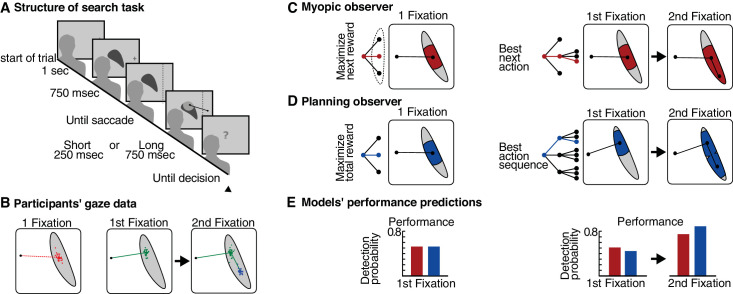
Difference between myopic and planning model. (A) Structure of the visual search task. In one condition, participants are allowed to make a single gaze shift (short), while in a second condition, participants are able to carry out a sequence of two gaze shifts (long). (B) Human gaze targets in the two conditions. (C) A myopic or greedy model of eye movement selection always selects the next best target. This corresponds to an independent sensory measurement, followed by a single decision, which in turn is followed by the actual gaze shift. (D) If instead eye movements are planned, then the sequences of two eye movements are selected in a way to ensure task success over the entire action sequence. This affects the first and second gaze shift. (E) In terms of detection performance, the myopic and planning models provide identical predictions if a single eye movement is carried out. However, the planning model incurs a price in detection probability for the first gaze shift, but the overall sequence achieves a higher expected detection probability over the behavioral sequence.

The experiment consisted of a visual search task in which subjects searched for a hidden target (see Figure 3A). The potential target, which was present in half of the trials, was highly visible, embedded in a noise pattern within an irregularly shaped region, large enough so that it could not be fully inspected with a single fixation. At the beginning of a trial, the subject maintained fixation until the search region was presented and then initiated the search to find the potential target by making an eye movement toward the shape. By monitoring gaze, it was possible to limit visibility to a region of radius 6.5° of visual angle around the fovea. The experimental manipulation across two conditions controlled whether subjects had one or two saccades at their disposal for finding the potential target. Thus, while in one condition, participants had to select a single gaze target and then decide whether the target was present, in the second condition, subjects could carry out two gaze shifts to find the potential target. The stimuli were semi-automatically designed to adjudicate between planning and greedy (i.e., nonplanning strategies).

A greedy gaze selection strategy carries out a single perceptual inference and, based on this inference, carries out a single next action to maximize the immediate expected reward. Then, the cycle starts anew by making a perceptual inference at the new gaze location and carrying out the next eye movement (Figure 3C). Thus, this strategy corresponds to a repeated, independent application of statistical decision theory, which is identical to the conceptualization of most psychophysical experiments. Such decision rules are common; for example, in saliency models, the next most salient gaze target (Itti & Baldi, 2005) is selected, and similarly, in “maximum a posteriori searchers,” the target that currently has the highest probability of being the target is selected (Eckstein, Thomas, Palmer, & Shimozaki, 2000). Similarly, the “ideal searcher” (Najemnik & Geisler, 2005), which selects the gaze location that is currently expected to yield the highest probability of finding the target after the single next gaze shift, also plans one gaze shift at a time. By contrast, a planning strategy considers a sequence of actions and therefore does not select the next best single gaze target from all possible gaze movements but selects the best sequence of gaze targets. For the experimental condition with two gaze shifts, a planning strategy jointly considers all possible first gaze targets together with all possible subsequent second gaze targets within the shape promising to maximize the probability of detecting the search target (Figure 3D). Note that this planning also includes the expected, probabilistic motor error associated with these gaze shifts (i.e., it takes into account that a planned gaze shift may not land exactly where it was targeted). The planning model then selects the single two-step gaze sequence that promises on average to provide the highest probability of detecting the target. This is because a sequence of two eye movements can spread out the gaze targets so as to cover more area of the entire irregular shape and thus maximize the probability of detecting the target.

The two strategies make predictions not only about the locations of gaze targets but also about task performance (see Figure 3E). When subjects plan sequences of eye movements, they choose gaze targets that take into account their future gaze targets. This is the means by which the performance over the entire sequence is evaluated. The consequence of this is that the first gaze shift in a sequence of two may seem “suboptimal” in the sense that the detection probability after a single gaze shift is smaller compared to the gaze target when a single eye movement is carried out.

#### The value of the computational model

The value of the computational POMDP planning model was not only that it allowed prediction of the observed gaze behavior across the two experimental conditions but that it accurately predicted the exact position on the different shapes where gaze should land, in close agreement with participants’ behavior (see Figure 3B). Furthermore, because the components of the POMDP model are readily interpretable in terms of uncertainty, variability, and costs, the authors extended both the planning and nonplanning models to incorporate additional biological constraints, that is, additive costs for longer saccades (Baloh, Sills, Kumley, & Honrubia, 1975), decreased peripheral acuity due to foveation (Geisler & Perry, 1998), and the empirical undershoot of human saccades (Harris, 1995). In each comparison, the planning model accounted better for the empirical data, and all planning models accounted better for the human gaze data than the best-performing greedy gaze selection model.

Given that the model can predict when subjects should plan sequences of eye movements, it allows future research asking how many gaze shifts human may plan ahead under which task circumstances, how planning costs trade off with task achievement, and what the conditions are in which eye movements are planned in the first place. For example, situations where two pieces of information are needed to make a decision might often reveal planning, especially when it is possible to save time with a preprogrammed sequence. It might also be useful to compare with planning of body movements, where planned sequences typically reduce energetic costs (Darici & Kuo, 2023). Thus, we now have a computational tool that can be used for more complex situations that arise in natural behavior. Although the task may seem different from a natural task, such as preparing a sandwich or making tea, and the model for selecting two subsequent gaze targets has already a high computational cost, the elements of the planning model address several of the common elements of natural visually guided behavior.

### Navigation as path-planning under uncertainty

**Figure 4.**
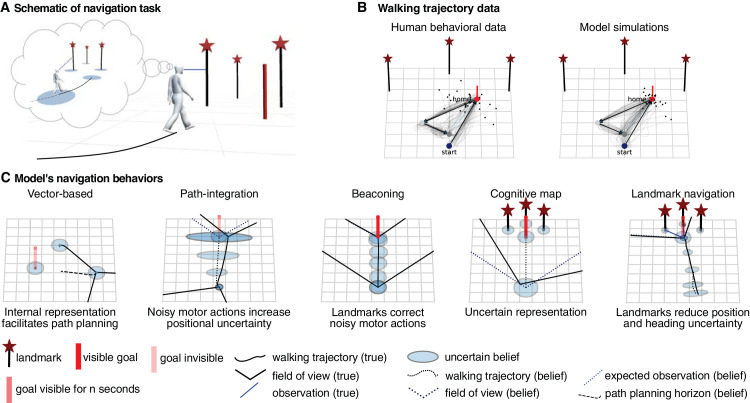
Navigation in the triangle completion task as path-planning in the framework of optimal feedback control under uncertainty. (A) Subjects maintain an internal, uncertain map relative to which they plan to navigate a triangular-shaped outbound path by picking up a sequence of three objects in an environment with landmarks prior to returning back to the location of the first object. (B) behavioral data and model simulations for the triangle completion task. (C) Depending on the relative uncertainties of sensory measurements, internal map, and the accrual of motor variability, optimal feedback control under uncertainty displays different navigational strategies described in the classic literature on navigation, including vector-based navigation, path integration, beaconing, navigating according to cognitive map, and landmark-based navigation.

One of the most fundamental behavioral tasks for humans and animals is moving from one point in space to a goal location. A well-studied experimental paradigm in a laboratory setting is the triangle completion task (Loomis et al., 1993), in which subjects navigate a triangular-shaped outbound path by picking up a sequence of three objects in an environment with landmarks, prior to returning back to the location of the first object. The advantage of this task is that it allows for the control of task parameters, which is not possible when studying the navigation of rugged terrain. Perhaps surprisingly, just manipulating the relative reliabilities of different navigational cues has been sufficient in past research to elicit an array of different navigational strategies, including beaconing, path integration, landmark-based strategies, strategies based on route knowledge, or the use of a cognitive map. How can this complexity in behavior be understood? Navigation is inherently a sequential visuomotor behavior, which unfolds over space and time and requires the actor to continuously and dynamically integrate sensory uncertainty about the positions of objects, landmarks, and the self in space, its internal cognitive map’s uncertainty, motor variability, and behavioral costs to plan subsequent actions. Kessler, Frankenstein, and Rothkopf (2024) formulated a dynamic Bayesian actor model of navigation as a POMDP, specifically in the framework of optimal feedback control under uncertainty. In the optimal control formalism, the necessary and sufficient variables for fully describing the momentary situation, the state variables, in this task comprise the position, heading direction and velocity of the walker, and the positions of the landmarks and targets. The changes of these state variables over time are fully specified by a dynamical system that describes how all state variables evolve over time, influenced by the movements of the walker. Classic optimal control under uncertainty assumes all involved noise distributions to be normal, allowing optimal behavior to be computed in closed form. Kessler, Frankenstein, and Rothkopf (2024) extended this framework to implement movement variability with signal-dependent noise, perception with state-dependent noise, and memory with time-dependent noise. The model furthermore assumes that humans continuously and sequentially plan their navigation actions based on subjective, internal beliefs combining different, uncertain sources of information. Thus, the planning is relative to the internal map and not relative to the physical, true space, which is never directly accessible (see Figure 4A). The model accounts for state estimation (Where am I? Where is my goal?), learning (What is the layout of the environment?), and path-planning and control (Where should I go? How do I get there?) while taking uncertainties in perception, internal representations, and action into account. Because all the relevant uncertainties interact continuously, the walker’s actions will not only change the distance to a target but also influence the relative uncertainties.

Importantly, because planning is based on internal, uncertain representations of the locations of actor, targets, and landmarks, planning a walking trajectory takes into account getting closer to not only the estimated target position but also the dynamic evolution of uncertainties. Thus, a plan for a walking trajectory balances reaching the goal with actively shaping the evolution of relative uncertainties of beliefs along the way. This is the fundamental reason why perception and action are inseparably intertwined in natural sequential behavior, and this constitutes the computational basis of active vision (Yang, Wolpert, & Lengyel, 2016). As Kessler, Frankenstein, and Rothkopf (2024) show, this behavior is captured by a POMDP model of navigation, which readily displays the diverse set of navigation strategies reported in previous literature (Figure 4C). Furthermore, with a single set of parameters, this model quantitatively predicts the rich and diverse sets of walking trajectories, navigation errors, and variability of walking endpoints (Figure 4B) in a large number of experiments and conditions from three previously published studies carried out in three different laboratories (Nardini, Jones, Bedford, & Braddick, 2008; Zhao & Warren, 2015; Chen, McNamara, Kelly, & Wolbers, 2017).

#### The value of the computational model

Beyond providing a quantitative account of human navigational behavior in triangle completion tasks, this computational model allows the resolution of previous contradictory results that seemingly either confirm (Nardini et al., 2008) or contradict (Zhao & Warren, 2015) Bayesian cue integration accounts, because it shows how sensory uncertainties interact with internal uncertainties and motor variability through path-planning. Similarly, the navigational strategies previously described in the literature are readily explained as arising from the uncertainties of different navigational cues relative to the uncertainty of the internal cognitive map. Thus, because this model of human navigation captures and explains a wide variety of data, it may be general enough to transfer to other navigation tasks, particularly because the model’s parameters are interpretable and accessible experimentally. For example, the scaling of the perceptual uncertainty of the target location as a function of distance and direction to the observer can be estimated using the model, as can the spatial variability of a single step. The model therefore opens up the possibility of quantitatively investigating open questions about human navigation (Savelli & Knierim, 2019), including questions such as how manipulating the reliability of visual cues might affect the selection of navigational strategies, how manipulation of the costs for movements might affect path planning, and how individual differences in navigation can be explained quantitatively in terms of perceptual uncertainty versus memory capacity.

### Explaining flexible strategies in catching a flyball

One of the limitations of laboratory experiments is that it is difficult to know how to generalize the results across contexts, experiments, or tasks. While the experiments discussed above used experimental designs involving several common elements of natural behavior, it is not clear whether the formal approaches used in those studies are also applicable to natural behavior in the wild. The interception of moving targets and particularly the catching of fly balls is a good test case, because ample research has described behavior across many different experimental conditions. Catching behavior has been described with several differing strategies or heuristics (Fooken, Kreyenmeier, & Spering, 2021), including Chapman’s theory consisting of the optic acceleration cancellation heuristic (OAC) and the constant bearing angle heuristic (CBA) (Chapman, 1968), the generalized optic acceleration cancellation theory (GOAC) (McLeod, Reed, & Dienes, 2006) and the linear optical trajectory theory (LOT) (McBeath, Shaffer, & Kaiser, 1995). These models identify lawlike rules that relate geometric quantities or their rates of change, such as the running direction to the direction toward the ball. How can these different strategies be explained, and why do they arise? And, if these different behaviors are conceptualized as different rule-like heuristics, on what basis do human decide how to switch between them? Belousov, Neumann, Rothkopf, and Peters (2016) provided a different approach by devising a computational model of catching as stochastic optimal feedback control in a continuous POMDP. These types of models have a long tradition in explaining human behavior in motor control (Wolpert & Ghahramani, 2000; Todorov & Jordan, 2002; Shadmehr & Mussa-Ivaldi, 2012), for example, in trial-based tasks with single movements of short duration, such as reaching and pointing. However, in their theoretical study, Belousov et al. (2016) applied this framework to the sensorimotor interception task and devised a model in which the running direction and the looking direction are jointly controlled so as to catch the ball with high probability. This allowed them to ask whether the running trajectories and the gaze behavior of an outfielder intercepting a flyball can be understood as a sequence of decisions under sensory uncertainty, internal model uncertainty, and motor variability.

First, in a common laboratory experiment, a small number of variables describe the stimulus (e.g., contrast or orientation) and the subject’s decisions (e.g., button pressed or not). When describing the catching of the flyball with optimal control under uncertainty, the continuous state variables are the positions and velocities of both the outfielder and the ball in space. The outfielder is additionally characterized by the gaze direction vector. The changes of these state variables over time are again fully specified by a dynamical system that describes both how the objects’ state variables evolve over time (e.g., the position and velocity of the ball along its trajectory) and how actions (i.e., the actions, or control, exerted by the outfielder both as the direction of running and the direction of gaze) affect all state variables. However, different from the common reinforcement learning setting, the outfielder does not have access to the true state of the world, because sensory observations are inherently uncertain and, moreover, delayed in time. This is where stochastic optimal control assumes that the actor obtains sensory measurements from the world, formalized through the observation function, and that the actor maintains an internal, probabilistic belief state about the most likely state of the world based on the sequence of sensory observations. Thus, the outfielder does not know the true, physical positions and velocities of the ball and of himself, but using his sensory observations and internal model of the dynamics of the world, he maintains the most likely positions, together with their uncertainty in an internal belief state. In the model proposed by Belousov et al. (2016), this belief is computed by approximate sequential Bayesian inference using the extended Kalman filter.

**Figure 5.**
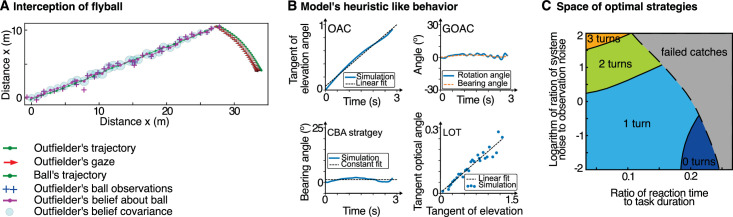
Ball catching as optimal feedback control under uncertainty. The optimal feedback control under the uncertainty model of ball interception can be used to simulate an outfielder catching a flyball (Belousov et al., 2016). (A) The optimal control law describes both how the outfielder should run to catch the ball and in which direction he should look. For balls flying toward the outfielder, gaze can be maintained on the ball. Note that the model also computes the internal uncertain beliefs about the position of the ball. (B) All previously described heuristics (i.e., the OAC strategy, the constant bearing angel heuristic, the tracking heuristic as part of the GOAC strategy, and the LOT strategy) are all fulfilled, despite the fact that the model neither measures nor represents any of the angles implied by the heuristics. (C) For long passes coming from behind the outfielder, the model additionally predicts the switch between different catching behaviors, specifically, how many times the outfielder turns his head between looking in the direction of locomotion versus in the direction of the flyball.

Formulating the outfielder problem in this way allows computing an approximate optimal feedback control policy, that is, a sequence of movements of both the outfielder’s body and eyes (see Figure 5A), which on average maximize the probability of catching the flyball in the end. Of course, from everything that is known about perception and motor control, estimates of the position and speed of both the ball and the outfielder himself in space are highly uncertain. Similarly, the internal predictions of the ball and motor control by the outfielder additionally introduce variability. This is the reason why predicting the ball’s trajectory according to the laws of physics and simply walking to the predicted point of contact would be a highly uncertain strategy, which is therefore not adopted. Importantly, because gaze direction can be controlled, a trade-off arises between directing gaze toward the ball to reduce uncertainty about its position and velocity, as well as looking in the direction of one’s own movement, to reduce uncertainty about where one is headed. This is a direct consequence of the increased uncertainty in the periphery, which is part of the optimal control model.

#### The value of the computational model

Belousov et al. (2016) showed that the four main heuristics described in the literature, the optic acceleration cancellation (OAC) heuristic, the constant bearing angle (CBA) heuristic, the LOT strategy, and the GOAC strategy, are optimal solutions if the catcher has sufficient time to continuously visually track the ball (see Figure 5B). This is a remarkable result as the optimal control under the uncertainty model neither observes nor represents any of the angles that had been identified as potential variables that the outfielder would observe to catch a flyball. Moreover, optimal control under uncertainty finds an explanation for switches between different catching behaviors. The optimal coordination of running and gaze direction varies with the time available to catch the ball relative to the catcher’s sensory delay, as well as the sensory uncertainty relative to the uncertainty in the predictability of the flyball’s trajectory. Specifically, by varying model parameters such as noise, time to ground contact, and perceptual latency, it is shown that different strategies arise under different circumstances. The outfielder’s policy switches between generating reactive and predictive behavior based on the ratio of system to observation noise and the ratio between reaction time and task duration (see Figure 5C). Evidence for the latter explanation was later found across a large number of experiments on manual interception (Fooken, Kreyenmeier, & Spering, 2021).

Thus, this study provides a rational account of human ball-catching behavior and a unifying explanation for seemingly contradictory theories of target interception on the basis of stochastic optimal control. Moreover, the model opens up the possibility of investigating whether the lawlike interception heuristics can also account for catching behavior in specific situations, such as when the ball approaches from behind and flies over the subject’s head. In this situation, the POMDP model predicts subjects would switch between looking in the direction of locomotion and looking toward the ball during a catch, which is different from the heuristics’ behavior and could therefore disambiguate the explanations for ball-catching behavior.

## Moving forward: Inverse modeling of natural behavior

The examples described above suggest that laboratory experiments can be designed to elicit common elements of natural visually guided behavior if their statistical structure is chosen appropriately. For example, the temporal event detection task was designed to involve perceptual uncertainties about the duration of events and allowed learning of event priors over trials. Therefore, the event probability changes moment-by-moment at the two locations. Accordingly, carrying out an eye movement in this task is not driven by low-level image properties (i.e., the visual stimulus) but is instead determined by the moment-to-moment changes in internal beliefs of the subject. This is much closer to situations encountered in natural behavior, such as making a saccade when predicting a pass in sports or when checking the walking direction of pedestrians while crossing a busy street. The examples also suggest that some progress can be made by devising laboratory experiments that involve common elements of natural behavior but allow for a higher degree of control (e.g., replacing single, short-duration, independent trials; deterministic states; and action outcomes with extended sequential actions in longer-duration behavioral sequences with sensory uncertainty and probabilistic action outcomes). These example studies reviewed above also show that behavior in such experiments is becoming amenable to computational modeling, and therefore to a deeper understanding of the components of natural sensorimotor behavior. After each of the studies described, we have explicitly outlined ways that the models and paradigms might be used to develop further insights over a broader range of natural behavior than that described in a single experiment.

As seen in the previous examples, such tasks may be formalized in the framework of sequential decision-making under uncertainty as POMDPs. The overarching goal is to demonstrate that when sensory uncertainty, internal uncertainties in beliefs about the world, action outcome uncertainties, and costs and benefits of actions are considered jointly in sequential tasks, many common behavioral elements, including trade-offs between perception and action, switching between strategies, active sensing, and active learning, become readily explainable. The fundamental problem that needs to be solved is how to construct a bridge between the above laboratory experiments, in which stimuli and their uncertainties are fully controlled, and natural tasks, where it is unclear how to quantify statistical regularities of the task, the subject’s internal uncertainties about task-relevant quantities, and participants’ subjective utilities. Thus, the fundamental difficulty that needs to be overcome is how to infer quantitatively the models’ parameters directly from observed behavior. For example, what is the uncertainty of an event in natural tasks? What is the cost for making an eye movement when walking a rugged terrain, preparing a sandwich, or crossing a busy street? And, what is the uncertainty about the distance to a foothold when looking at it, and how does the cost of an eye movement depend on the degree of ruggedness or steepness of terrain, or the angular distance between the direction of locomotion and the direction to a foothold, if at all? Classical trial-based psychophysics, together with signal detection theory, can only answer some of these questions.

Recent advances in machine learning nourish the hope that the unknown internal subjective parameters in sequential naturalistic behavior, such as the behavioral costs or subjective utilities, can be recovered directly from behavioral data. One broad class of such methods, called inverse reinforcement learning, assumes that the underlying task from which observed behavior stems can be formalized as a Markov decision process (MDP). Under these assumptions, inverse reinforcement learning methods recover the rewards or costs from observed behavioral data alone. Originally developed in machine learning (Ng, Russell et al., 2000), such methods have been adapted to account for human behavior, for example, locomotion (Mombaur, Truong, & Laumond, 2010) and spatial navigation (Rothkopf & Ballard, 2013), table tennis (Muelling, Boularias, Mohler, Schölkopf, & Peters, 2014), but also attention switching (Schmitt, Bieg, Herman, & Rothkopf, 2017) and task prioritization (Zhang et al., 2018). These methods explain deviations from “optimal” behavior in a task by inferring subjective costs or rewards underlying human behavior in terms of cognitive constraints and uncertainties of the internal beliefs about the world state. More recently, inverse reinforcement learning has been further extended to inferring the costs as well as other quantities, such as the perceptual uncertainties, action variability, or even an agent’s internal model of a task implicit in the observed behavior. Different from inverse reinforcement learning, these methods now explicitly also address perceptual uncertainty and, accordingly, invert POMDPs. This class of methods, also originally developed in machine learning (Choi & Kim, 2011), comprises a variety of different algorithms that are distinguished by the respective assumptions, such as whether time is modeled as discrete or continuous, whether the observed actions are corrupted by noise, or whether the noises are Gaussian or following some other distribution.

Of particular interest for inferring the unknown quantities in human natural visually guided behavior are methods that consider continuous state variables that can thus accurately describe continuous movements of the body and of objects, such as the catching of flyballs or the walking toward goals in spatial navigation. Such methods have been developed recently, both under the assumption of generic Gaussian noise (Kwon, Daptardar, Schrater, & Pitkow, 2020) but also incorporating the known human uncertainties and variabilities (Schultheis, Straub, & Rothkopf, 2021), that is, signal-dependent motor variability (Jones, Hamilton, & Wolpert, 2002) and Weber-law phenomena in perception. And finally, recent methods for inverting POMDP models have been developed that can disentangle whether an observed action was carried out to reduce uncertainty or for getting closer to the goal (Straub, Schultheis, Koeppl, & Rothkopf, 2023), that is, the specific case in which perception, cognition, and action are intertwined, such as in goal-directed sequential behavior involving active vision. In a first application to the experimental paradigm of continuous psychophysics, Straub and Rothkopf (2022) showed that such methods can successfully recover perceptual uncertainty, motor variability, internal costs, and even the false beliefs that individual subjects have about experimental parameters. However, such methods have not yet been applied to extended natural tasks, and it is currently an open question whether these methods scale to such complex data sets.

In conclusion, the goal of this article was to address the problem of how to study visually guided actions in the natural world, given the recent calls for such research. We pointed out that, despite the diversity and scope of natural behavior, there is, in fact, a lot of commonality over a broad range of everyday visually guided activity. By devising experiments that respect this commonality, while explicitly investigating one aspect of the *behavior*, we can gain some traction in understanding the underlying visuomotor control mechanisms and properties. This effort gains power from using models that reflect the complexity of the sequences of decisions involved in even the simplest natural behaviors. Taken together with methods for estimating the unobserved cognitive quantities that determine behavior, the hope is that we can reach a deeper understanding of the control of visually guided actions.
